# Tuberculosis of the Spine in a Patient With Kidney Cancer

**DOI:** 10.7759/cureus.36427

**Published:** 2023-03-20

**Authors:** Irena Karabella, Efstathios Chronopoulos, George Panagiotakopoulos, Ismene A Dontas

**Affiliations:** 1 Infectious Diseases, Sotiria Thoracic Diseases Hospital of Athens, Athens, GRC; 2 Orthopaedic Surgery, Laboratory for Research of the Musculoskeletal System, KAT General Hospital, Medical School, National & Kapodistrian University of Athens, Athens, GRC; 3 Clinical Pharmacology/Pharmacology, University of Patras, Patras, GRC; 4 Veterinary Medicine, Laboratory for Research of the Musculoskeletal System, KAT General Hospital, Medical School, National & Kapodistrian University of Athens, Athens, GRC

**Keywords:** lesions, metastasis, kidney cancer, spine tuberculosis, m. tuberculosis

## Abstract

Tuberculosis is a widespread, airborne infectious disease caused by *Mycobacterium tuberculosis* bacteria. This infection is often misdiagnosed, particularly in the case of spinal tuberculosis which can present atypically. Although rare, tuberculosis can mimic bone tumors or metastatic lesions in the spine. In patients with *immunosuppression* who have cancer and show signs of lymph node involvement and bone lesions, it is important to explore other potential causes as part of the diagnosis process. Here, we present a case of renal cell carcinoma in which a lytic lesion in the spinal cord was initially misdiagnosed as a metastatic lesion due to the presence of cancer. Skeletal tuberculosis is a rare condition, and it is crucial to maintain a high level of suspicion for a proper diagnosis.

## Introduction

Tuberculosis is transmitted by airborne infectious aerosol, caused by Mycobacterium tuberculosis (TB) bacteria. TB is capable of infecting any human organ, starting with the lung, usually as a reactivation years later or at the same time as the primary infection, which can lead to hematogenous dissemination [1].

People at high risk of developing TB disease are those who were born or have lived in countries with a high incidence of tuberculosis due to an increased risk of latent TB infection [2]. Skeletal infections occur when TB spreads to bones and joints through vascular channels [3]. The clinical presentation of skeletal tuberculosis can be serious and can lead to severe consequences due to the frequent delay in diagnosis caused by its nonspecific symptoms [4]. Active TB is most commonly found in the lung, while bone tuberculosis and joint tuberculosis are rare in extrapulmonary infections. In immunosuppressed patients with malignancy who present with lymph node involvement and bone lesions, it is crucial to consider other causes as a differential diagnosis.

## Case presentation

A 63-year-old male patient presented to the emergency department with a low-grade fever of up to 37.6^ο^ C and a nine-month history of worsening low back pain when walking. His past medical history was renal cell carcinoma on the right kidney which was treated surgically and with systemic therapy a year prior.

On clinical examination, the patient was hemodynamically stable with a blood pressure of 105/80 mmHg and a pulse rate of 60 beats per minute. He presented with decreased lung expansion and diminished breath sounds, but he was asymptomatic (oxygen saturation (SatO_2_) was 97% (FiO_2_ 21%)). An expiratory paresis and respiratory depression were noted on the right hemithorax. The abdomen was soft and painless, with bowel sounds present. The patient was awake and alert, with a Glasgow scale score of 15/15. There were tenderness and a positive Lasegue's sign upon examining the lumbar region. Muscle strength was 5/5 in the upper extremities and 4/5 in the lower extremities. The superficial and deep tendon reflexes were normal. The patient had elevated levels of inflammatory markers, without concomitant lymphocytosis, and also elevated values of cholestatic enzymes (Table 1).

**Table 1:** Blood test results of the patients at initial medical examination.

Lab Tests	Values	Normal Values
Erythrocyte Sedimentation Rate	80 mm/hr	< 20 mm/1hr
C-Reactive Protein	62 mg/L	< 5 mg /L
Alkaline Phosphatase	200 IU/L	25-125 IU/L
Gamma-Glutamyl Transferase	147 IU/L	10-49 IU/L
Bilirubin	1.3 mg /0.6 mg	0.3-1.2 mg

During his hospital stay, the patient experienced intermittent episodes of fever, leading to blood and urine cultures being taken, but all results were negative. A previous imaging scan, consisting of an upper and lower abdominal computed tomography (CT) scan performed a year ago, showed an osteolytic lesion at thoracic 12 vertebrae (T12), along with a synchronous soft tissue lesion in the anteroposterior and paraspinal space, which is consistent with a secondary location.

Based on the previous findings, tests for Brucellosis (Wright and Wright-Combs), serum angiotensin-converting enzyme, and Quantiferon (+) were performed. An additional CT scan of the chest was conducted, revealing an encapsulated lateral collection on the right measuring 9.4 x 6.4 cm, as well as inhomogeneity and structural disturbances in the architecture of the vertebrae T11-T12, with bone erosions, lytic changes, and a wedge-shaped deformation of T12, along with accompanying changes in the density of the paraspinal soft tissue. These findings are consistent with spondylodiscitis.

Fine-needle aspiration of the lateral collection and paravertebral mass was performed, and initial testing using Ziehl-Neelsen staining and rRNA Mycobacterium tuberculosis molecular detection was negative. However, due to a high suspicion of tuberculosis, the patient was started on a two-month course of anti-tuberculosis treatment consisting of isoniazid, rifampicin, ethambutol, and pyrazinamide. The culture eventually came back positive and showed no resistance to rifampicin and isoniazid.

**Figure 1:**
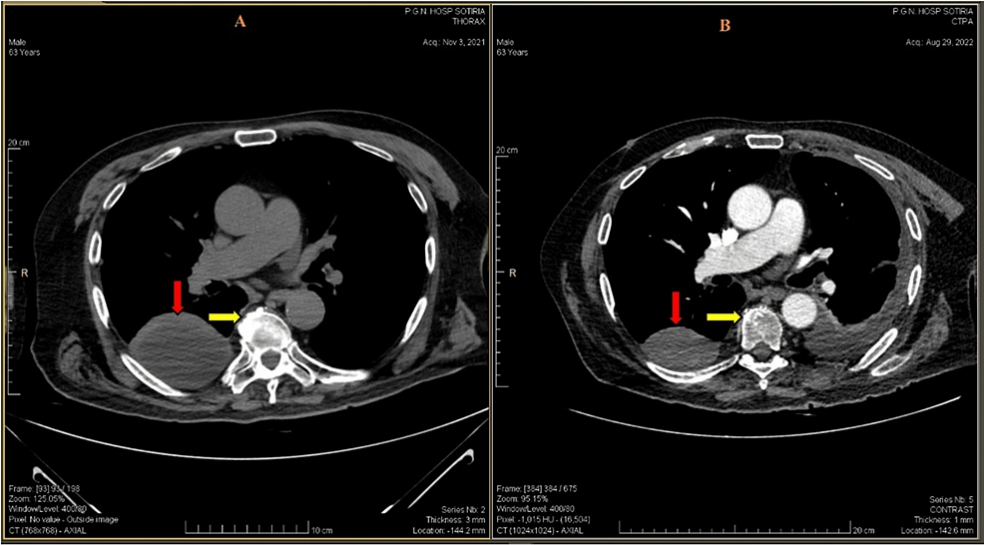
Thorax CT scans. A) Pre-treatment imaging shows a Th12 spinal lesion (yellow arrows) and paraspinal fluid collection (red arrows) and B) imaging nine months after treatment indicates a decrease in the spinal lesion (yellow arrows) and paraspinal fluid collection (red arrows) CT: Computed tomography

As part of the follow-up to treatment, a new lumbar and thoracic spine magnetic resonance imaging (MRI) was performed and showed improvement in the architecture of T11-T12, as well as in the peripheral soft tissue surrounding the vertebrae, with areas of ossification in the bodies. After completing the two-month course of treatment, the patient continued to receive a two-drug therapy regimen of isoniazid and rifampicin. After 10 months, a repeat chest CT scan showed further improvement (Figure 1).

## Discussion

Tuberculosis of the spine, also known as spinal tuberculosis or Pott's disease, can occur in patients with kidney cancer. It is a serious condition that requires prompt and appropriate treatment.

Clinical presentation of spinal tuberculosis may include low back pain that worsens with walking, fever, and loss of weight. Radiological findings may resemble malignancy, leading to a delay in diagnosis. In such cases, a fine needle biopsy or a culture of the affected tissue may be necessary to confirm the diagnosis [5].

The treatment of spinal tuberculosis in patients with kidney cancer requires a combination of anti-tuberculosis drugs, often given for 6-9 months, along with surgery in certain cases. It is important to closely monitor the response to treatment, as well as any potential side effects or drug interactions with other medications used for the treatment of kidney cancer.

In conclusion, spinal tuberculosis can be easily misdiagnosed as metastatic bone cancer due to its nonspecific presentation and imaging findings. The destruction of vertebral bodies and associated paravertebral abscesses in spinal TB can mimic bone metastases on imaging, leading to misdiagnosis. Early recognition and appropriate management are crucial for a good clinical outcome [6].

In the present case, the patient’s previous imaging scan showed an osteolytic lesion at the Th12 vertebra of the spine, along with a concurrent soft tissue lesion, which was suspicious for a spinal tumor (Figure 1A). The CT-guided fine-needle aspiration biopsy confirmed the mass as a tuberculoma. However, the existence of bone destruction and a soft tissue mass without calcification in some areas remains a matter of debate. The biopsy taken from the spine lesions did not reveal any signs of malignancy and resulted in a diagnosis of chronic inflammation. A thoracic and lumbar MRI revealed a solid mass resembling a malignant spinal image. Radiologic indications for a tuberculosis diagnosis, as well as the pattern of rim enhancement in the soft tissue masses of the spine, can be seen in images of multiple lymph node pathologies. According to available literature, both the location of the area of concern and the presence of a central hypodense area are key factors in making a diagnosis.

## Conclusions

Radiological findings of skeletal tuberculosis can resemble those of malignancy, which can result in a delayed diagnosis. Furthermore, extrapulmonary tuberculosis can cause more pronounced symptoms of the disease, with or without the presence of minimal pulmonary tuberculosis. Spinal tuberculosis and metastatic cancer are commonly diagnosed lesions in the spine. Bone metastases are frequently observed in patients with various primary cancers. It can be challenging to distinguish between clinical presentations of TB and metastasis, especially if the typical signs of TB are not apparent. Thus, it is important for healthcare providers to consider TB in the differential diagnosis of patients with suspicious findings on imaging tests, especially in high-risk populations. Prompt recognition and treatment of TB can help prevent complications.
